# Affective Temperaments and Meteoropathy Among Women: A Cross-sectional Study

**DOI:** 10.1371/journal.pone.0232725

**Published:** 2020-05-04

**Authors:** Włodzimierz Oniszczenko

**Affiliations:** Faculty of Psychology, University of Warsaw, Warsaw, Poland; Universita Cattolica del Sacro Cuore Sede di Roma, ITALY

## Abstract

The main goal of the study was to assess the relationship between affective temperaments and meteoropathy among women and examine meteorosensitivity as a mediator in this relationship. The issue of affective temperaments and meteoropathy has not been considered in the literature. The sample consisted of 450 Caucasian women gathered via the online recruitment platform. The participants’ ages ranged from 18 to 70 years (M = 30.01; SD = 9.10). The Polish version of the Temperament Evaluation of Memphis, Pisa, Paris and San Diego Autoquestionnaire was used to assess affective temperaments (depressive, cyclothymic, hyperthymic, irritable and anxious). Meteorosensitivity and meteoropathy were assessed using the Polish adaptation of the METEO-Q questionnaire. A large positive correlation was found between meteorosensitivity and meteoropathy. Medium positive correlations were found between meteorosensitivity/meteoropathy and cyclothymic and anxious temperaments. Small positive correlations were revealed between depressive and irritable temperaments and both meteorosensitivity and meteoropathy scales. No correlation was found between hyperthymic temperament and meteorosensitivity/meteoropathy. Mediation analyses indicated cyclothymic and anxious temperaments affected meteoropathy both directly and indirectly through meteorosensitivity as a mediator. The most severe meteoropathy symptoms in the studied sample were asthenia, an indefinite feeling of malaise and irritability. The results suggest affective temperaments may be related to meteoropathy symptoms in women.

## Introduction

People are warned of the effects of changing weather on TV and through online messages every day. Every day, some people report their health deterioration or some new symptoms they associate with changes in weather—such as temperature, humidity, cloudiness or atmospheric pressure changes. This usually applies to women and older or middle-aged people, although it may also apply to children [1]. It is estimated that approximately 55% of the German population and 69% of the Canadian population over the age of 60 years seem to be weather sensitive—that is, they consider the weather affects their health [2].

Behavioral data suggest environmental factors such as climate or weather may impact human emotional states and health conditions. For example, it was demonstrated that emotional distress decreases when exposure to the sun increases [3] and during day-to-day weather variations (solar exposition, wind speed and air pressure influences self-reported life satisfaction [4] and low and high temperature levels, precipitation, humidity and cloud cover worsens the expression of feelings among social media users [5]. It was also demonstrated that environmental stressors—such as considerable increase in temperature or increased precipitation produced by climate change, especially during natural disasters—may cause mental, emotional and bodily stress among humans [6, 7]. Weather conditions are also related to seasonal affective disorder, which is predominantly found in women [8], and panic anxiety attacks [9]. Some authors also pointed out the relationship between weather changes and cluster headache [10] (Lee et al. 2014), migraine [11], neuropathic pain [12] and human sleep disturbance [13].

Any disorders resulting from the impact of climatic conditions can be described as meteoropathy [1]—that is, a phenomenon of worsening existing diseases or the emergence of a new specific disease as a consequence of climate change [14]. Meteoropathy includes “a group of symptoms and pathological reactions in response to gradual or sudden changes in meteorological factors in a specific area interacting, presumably, through natural electromagnetic influences covering a wide range of frequencies and amplitudes” [1, p. 46]. In general, people vary in their sensitivity to weather changes—although the most vulnerable to meteoropathy are women, middle-aged people and anxious and depressed individuals [1]. It is worth noting that women are usually indicated as more sensitive to weather conditions, as well as more easily susceptible to developing symptoms of physical and mental disorders in response to environmental changes [15, 16, 7]. Meteorosensitivity is the key concept here. Meteorosensitivity may be defined as “biological susceptibility to feel the effect of particular atmospherical events on [the] mind and body” [17, p. 103]. Meteorosensitivity is also related to the human psychophysical capabilities linked with everyday stress management.

Although the biological mechanisms of meteoropathy are unknown, in a recent study, it was suggested that superior vestibular nucleus activity may affect meteoropathy regulation in mice and probably in humans [18]. Furthermore, the activities of the hypothalamus and amygdala nucleus are suggested as significant in meteoropathy development [19]. The authors suggested electromagnetic waves directly affect the hypothalamus. Therefore, they indirectly enhance the secretion of the stress hormone, the adrenocorticotropic hormone, and decrease endorphin secretion—which may lead to increased anxiety, headache and other meteoropathic symptoms. The authors assumed both human psychophysical instability and susceptibility to weather changes causing stress through changes in the brain regulation of emotions becomes a factor that increases human susceptibility to somatic and mental disorders.

From a psychological point of view, cerebral mechanisms of emotion regulation and autonomic nervous system (ANS) functioning are useful for understanding individual differences in meteorosensitivity levels and meteoropathy symptom development. Meteorological factors and other stressors may cause a challenge to the human physiological balance. When the balance is difficult to maintain, the development of somatic symptom disorder may be observed [20].

Taking account that many meteoropathy symptoms—such as lability of mood, depression, anxiety or an indefinite feeling of uneasiness—are related to mood disorder symptoms [17], it is worth considering the role of affective temperaments to explain the differences in meteoropathy among women.

Affective temperaments (depressive, cyclothymic, hyperthymic, irritable and anxious) refer to a genetically determined, stable-across-lifespan and trait-related manifestations playing a fundamental role in the predisposition to mood disorders—such as depression, anxiety and bipolar disorder [21, 22, 23]. Several studies have showed women have higher levels of the depressive, cyclothymic and anxious temperaments than men [24, 25, 26]. These results are in line with the observation that mood disorders are approximately twice as prevalent in women compared to men [27].

Molecular genetics research has provided interesting data on the strong association between central serotonergic (the depressive, cyclothymic, irritable and anxious temperaments) and dopaminergic (the hyperthymic temperament) regulation in affective temperament development [28]. It is worth noting that several authors have pointed out the role of serotonin in human mood regulation [29, 30, 31]. Moreover, serotonin is involved in the hypothalamic–pituitary–adrenal axis reaction to acute and chronic stress, which contributes to the onset of anxiety and depression [32].

Affective temperaments may share a common biological disposition with various other psychiatric or somatic symptoms, including meteoropathic disturbances. For example, the cyclothymic/irritable temperament appears to be associated with elevated stress reactivity in daily life [33] and somatic symptoms with no organic explanation [34, 35]. In an earlier study, a strong association between the cyclothymic temperament and hypertension was demonstrated [36]. Certain affective temperaments, mainly the cyclothymic and anxious temperaments, have also been associated with sleep disturbances present among meteoropathic symptoms [37, 38]. In addition, the anxious temperament may be related to meteoropathy symptoms—such as anxiety, irritability, hypervigilance, inability to relax, insecurity, tension and gastrointestinal distress [28].

The main goal of this study was to assess the relationship between affective temperaments and meteoropathy among women and examine meteorosensitivity as a mediator in this relationship.

We hypothesized (a) the depressive, cyclothymic, irritable and anxious temperaments are positively correlated with meteoropathy, while the hyperthymic temperament is negatively correlated with meteoropathy and (b) the meteorosensitivity dimension mediates the relationship between affective temperaments and meteoropathy.

## Materials and methods

### Participants

The study sample consisted of 450 white women gathered from the general population via the University of Warsaw online recruitment platform. The participants’ ages ranged from 18 to 70 years (M = 30.01; SD = 9.10). Among the participants, 303 had received higher education, 146 had received secondary education and 1 had received primary education. Fifty-nine women lived in rural areas, 65 in small towns and 326 in large cities.

This was an anonymous cross-sectional study, participation was voluntary and the participants did not receive any compensation.

The data were collected via online self-report questionnaires. All participants provided informed consent to participate in the study after reading the rules of the study. Information about the purpose and procedure of the study was displayed on a computer screen. Starting the questionnaires was tantamount to agreeing to participate in the study on the given terms.

The research project including aforementioned procedure and all questionnaires used was approved by the local Research Ethics Commission at the Faculty of Psychology, University of Warsaw (ref: 5-03-2019).

### Measures

Affective temperaments were assessed using the Polish version of the Temperament Evaluation of Memphis, Pisa, Paris and San Diego Autoquestionnaire (TEMPS-A) [21, 39, 40]. TEMPS-A is a self-report instrument that is composed of 110 items (109 for men) with a yes/no response format. TEMPS-A is composed of five temperament scales (Cronbach’s alphas for the current sample are given in parentheses): depressive (α = .74), cyclothymic (α = .83), hyperthymic (α = .81), irritable (α = .84) and anxious (α = .88). For each answer, a “yes” was scored as 1 and a “no” as 0. These scores were added and then divided by the number of items that belonged to each affective temperament scale (21 for each of the first four temperament categories and 26 for the anxious temperament).

Meteorosensitivity and meteoropathy were assessed using the Polish adaptation of the METEO-Q questionnaire [17] (Polish adaptation by Włodzimierz Oniszczenko). The questionnaire consists of 11 items that measure meteorosensitivity (5 items) and meteoropathy (6 items) and a structured checklist aiming to identify the 21 physical and psychological symptoms related to climate variations (see Table 3). All items are rated on a 4-point Likert response scale ranging from 0 (absent) to 3 (severe). Cronbach’s alphas in the current sample are given in parentheses: meteorosensitivity scale (α = .79) and meteoropathy scale (α = .79).

### Statistical analysis

The statistical analysis was performed using IBM SPSS Statistics 25 [41]. Descriptive statistics, such as mean and standard deviation, of the main variables were recorded. Data normality was checked based on the skewness and kurtosis values (ranged from −1.5 to 1.5), following the application of criteria specified by Tabachnick and Fidell [42]. Relationships among variables were examined with Pearson product–moment coefficients. An absolute value of *r* of .1 was classified as small, .3 as medium and .5 as large per Cohen [43]. The mediation analyses were conducted using the PROCESS Model 4 macro for SPSS v. 3.3 [44]. In addition, the bootstrapping procedure with 5,000 sample draws and bias-corrected standard errors was used to estimate the direct and indirect effects [45].

## Results

Table 1 provides descriptive statistics, as well as skewness and kurtosis values, for meteorosensitivity, meteoropathy and affective temperaments for the whole sample. The skewness and kurtosis analyses show that all the variables were normally distributed.

**Table 1:** Descriptive statistics for the meteorosensitivity and meteoropathy and Temperament Evaluation of Memphis, Pisa, Paris, and San Diego Autoquestionnaire (n = 450).

Variable	Range	Mean	Standard deviation	Skewness	Kurtosis
*METEO-Q*					
Meteorosensitivity	0–15	9.49	3.11	-.54	.18
Meteoropathy	0–18	8.81	3.74	-.07	-.23
*Affective temperaments*					
Depressive	.00–.95	.44	.18	.26	-.52
Cyclothymic	.00–.95	.42	.22	.20	-.69
Hyperthymic	.00–1.00	.44	.21	.10	-.73
Irritable	.00–.95	.31	.21	.53	-.39
Anxious	.00–1.00	.42	.23	.26	-.58

Table 2 presents the correlation coefficients between age, meteorosensitivity, meteoropathy and affective temperaments in the studied sample. A small positive correlation was found between age and the hyperthymic temperament. Furthermore, small negative correlations were found between age and the cyclothymic, irritable and anxious temperaments. A large positive correlation was found between meteorosensitivity and meteoropathy. Medium positive correlations were found between meteorosensitivity and the cyclothymic and anxious temperaments, as well as between meteoropathy and the cyclothymic and anxious temperaments. Both the meteorosensitivity and meteoropathy scales positively correlated with the depressive and irritable temperaments (all the correlation coefficients were small). No correlation was found between mateorosensitivity and meteoropathy and the hyperthymic temperament.

**Table 2:** Pearson r correlations between age and meteorosensitivity and meteoropathy and Temperament Evaluation of Memphis, Pisa, Paris and San Diego Autoquestionnaire scales (n = 450).

	2.	3.	4.	5.	6.	7.	8.
1. Age	.03	.08	-.07	-.19***	.11**	-.15**	-.12**
2. Meteorosensitivity		.70***	.17***	.33***	.02	.26***	.31***
3. Meteoropathy			.18***	36***	.05	.27***	36***
4. Depressive				.56***	-.60***	.44***	.67***
5. Cyclothymic					-.21***	.67***	.61***
6. Hyperthymic						-.20***	-.43***
7. Irritable							.50***
8. Anxious							-

** p < .01;

*** p < .001.

**Fig 1:**
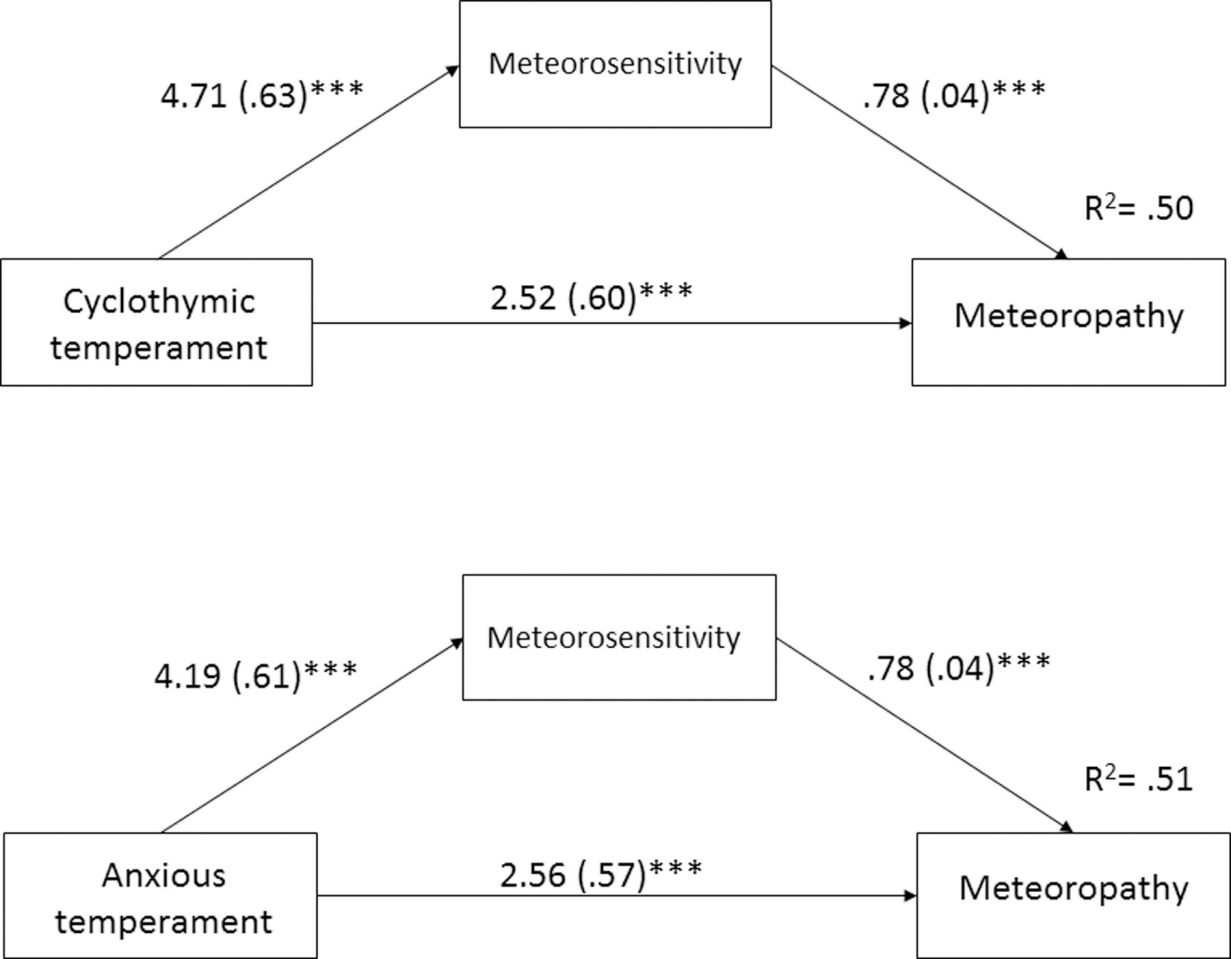
The mediating effect of meteorosensitivity in the relationship between the cyclothymic temperament and meteoropathy (upper) and the relationship between the anxious temperament and meteoropathy (lower). The unstandardized coefficients are reported, with standard errors in parentheses. The r-squared coefficients for the models are placed on the right side. *** p < .001.

Two separate analyses were performed using a bootstrapping procedure, with meteorosensitivity as the mediator between the cyclothymic and anxious temperaments and meteoropathy. The first analysis indicated a significant indirect effect of the cyclothymic temperament on meteoropathy through meteorosensitivity [effect = 3.65, SE = .53, 95% CI = (2.64, 4.73)]. The second analysis found a significant indirect effect of anxious temperament on meteoropathy through meteorosensitivity [effect = 3.25, SE = .50, 95% CI = (2.30, 4.26)]. The individual pathways in the mediation analysis are displayed in Fig 1.

Table 3 presents means and standard deviations for each meteoropathy symptoms provided by the METEO-Q questionnaire. As observed in Table 3, the most severe meteoropathy symptoms in the studied sample were asthenia, an indefinite feeling of malaise and irritability (75th percentile).

**Table 3:** Means and standard deviations for each meteoropathy symptom measured by the METEO-Q (n = 450).

	Range	M (SD)
Lability of mood	0–3	1.51 (.81)
Extreme reactivity to external events	0–3	1.37 (.93)
Depression	0–3	.94 (.98)
Anxiety	0–3	.78 (.91)
Asthenia	0–3	1.98 (.85)
Anhedonia	0–3	1.33 (.92)
Irritability	0–3	1.81 (.90)
Indefinite feeling of malaise	0–3	1.93 (.86)
Vague pain, articular pain, muscular pain	0–3	1.20 (1.09)
Vertigos	0–3	.83 (.94)
Headache	0–3	1.54 (1.05)
Nausea	0–3	.42 (.73)
Alterations of the cardiac rhythm (tachycardia)	0–3	.63 (.88)
Difficulties with concentration	0–3	1.46 (.93)
Insomnia	0–3	1.00 (1.03)
Excessive sleepiness	0–3	1.72 (1.00)
Lack of appetite	0–3	.55 (.78)
Excessive appetite	0–3	.87 (.98)
Digestion’s dysfunctions	0–3	.70 (.91)
Alterations of sexuality	0–3	.86 (.99)
Weakness during working activities	0–3	1.60 (.90)

## Discussion

The results of the present research may extend the knowledge of the functional significance of affective temperaments and indicate the importance of affective temperaments for women’s meteoropathy symptoms.

As expected, four affective temperaments (depressive, cyclothymic, irritable and anxious) were positively correlated with meteoropathy among women, although the correlation coefficients were small to medium. The medium correlations between the cyclothymic and anxious temperaments and meteoropathy were revealed. These results indicate the cyclothymic and anxious temperaments may be associated with some meteoropathy symptoms similar to mood disorder symptoms. It is worth noting that the most severe symptoms of meteoropathy in the studied sample were asthenia, an indefinite feeling of malaise and irritability. Meteorosensivity was strongly correlated with meteoropathy and served as a mediator between affective temperaments and meteoropathy among the studied women. This result is not surprising because the level of sensitivity to climate or weather changes (i.e., meteorosensitivity) seems to be the most important factor triggering the symptoms of meteoropathy. Therefore, one of the most important questions concerns the link between affective temperaments and meteorosensitivity and meteoropathy. The analysis of mediation shows the cyclothymic and anxious temperaments affect meteoropathy both directly and indirectly through meteorosensitivity as a mediator. The lack of sufficient data on the biological basis of both affective temperaments and meteoropathy does not allow the formulation of a clear hypothesis about the common biological mechanism of these phenomena.

Nevertheless, taking account of the hypothesis on the biological basis of affective temperaments, as well as the hypothetical biological foundations of meteorosensitivity, it can be assumed that some biological associations between these variables are related to brain activity and ANS functioning. The serotonergic regulation of the cyclothymic and anxious temperaments may connect affective temperaments with the so-called somatic awareness [26]. It is a physical discomfort that is medically unexplained, is associated with reduced levels of serotonin [46] and present in meteoropathy as, for example, headaches, fatigue or difficulty sleeping. Serotonin, involved in the functioning of the hypothalamic–pituitary–adrenal axis, can contribute to the development of two important meteoropathy components: anxiety and depression [32]. In a recent work, Di Nicola et al. [47] indicated that meteorological factors such as sunlight or atmospheric pressure are associated with changes in the level of serotonin in the brain, which—in addition to dysfunction in the dopaminergic system—may be important for the process of emotion dysregulation. In turn, Mazza et al. [48] suggested the increase in sympathetic nervous system tension resulting from a deficiency of serotonin metabolism in patients with fibromyalgia results in fatigue and weakness, sleep problems, headache and migraine, anxiety or depression and mood disorders. It is worth noting that these symptoms coincide with those that are present in meteoropathy.

The probable impact of cerebral mechanisms of emotion regulation and ANS functioning on meteorosensitivity and meteoroptahy is also visible in affective temperaments traits, such as an intense experience of all emotions or rapid changes in mood and energy typical for the cyclothymic temperament [21] or tension and gastrointestinal distress typical for the anxious temperament [39]. The ANS may be of particular importance for meteorosensitivity and the development of meteoropathy associated with the affective temperaments. The ANS functions penetrate the functioning of many other internal systems and organs, and dysfunctions of this system can manifest as various disorders of the body. It is worth noting, however, that the mechanisms combining affective temperaments with meteorosensitivity and meteoropathy and differentiating women from men can be much more complex and involve sex-dependent neurobiology and genetic, hormonal and immune functions, as well as sex-environment interactions, as suggested by some authors [49, 50].

Several limitations of this study should be acknowledged. This research is cross-sectional in nature, making it impossible to draw any definite conclusions regarding the direction of the relationship between affective temperaments and meteorosensitivity and meteoropathy. Affective temperaments and other personality traits may play a significant role in the development of meteoropathy symptoms, but further longitudinal studies are required to corroborate this hypothesis. Another important limitation of our study is the covariability of factors treated as predictors of meteoropathy symptoms. We only used data based on self-reports, and the presence of comorbid physical and mental health disorders or substance use was not documented. Therefore, the interpretation of our results is restricted. In addition, we observed only women, thus limiting the generalizability of the results. It should be added that the study sample was not too large when considering the survey in the general population.

## Conclusions

Regardless of the methodological limitations of our study, however, our results may contribute to the understanding of how psychological factors influence weather-related changes in women's well-being. Our findings highlight the significant overlap between affective temperaments and meteoropathic symptoms among women.

High levels of cyclothymic and anxious temperaments may be responsible for an increase in negative physical and mental health symptoms among women. To improve women’s well-being, meteorosensitivity and meteoropathy should be taken into account when assessing medically difficult-to-explain symptoms in women. Our results suggest that meteoropathic symptoms may be associated with moods and the dominant type of affective temperament in women. Thus, women who show mood lability and anxiety symptoms should be a focus of medical and psychological care.

## Supporting information

S1 Dataset (SAV)

## References

[1] Janiri L, Spinetti G, Mazza M, Di Nicola M. Meteoropathy. A new disease In: Christodoulou GN, Jorge M, Mezzich JE, editors. Advances in Psychiatry. Vol. 3 Athens, Greece: Beta Medical Publishers; 2009 pp. 45–52.

[2] Von Mackensen S, Hoeppe P, Maarouf A, Tourigny P, Nowak D. Prevalence of weather sensitivity in Germany and Canada. Int J Biometeorol. 2005; 49: 156–166. 10.1007/s00484-004-0226-2 15338386

[3] Beecher ME, Eggett D, Erekson D, Rees LB, Bingham J, Klundt J. Sunshine on my shoulders: Weather, pollution, and emotional distress. J Affect Disord. 2016;205: 234–238. 10.1016/j.jad.2016.07.021 27449556

[4] Feddersen J, Metcalfe R, Wooden M. Subjective wellbeing: Why weather matters. J R Stat Soc A Stat. 2016;179: 203–228. 10.1111/rssa.12118

[5] Baylis P, Obradovich N, Kryvasheyeu Y, Chen H, Coviello L, Moro E, et al Weather impacts expressed sentiment. PLoS ONE. 2018;13: e0195750 10.1371/journal.pone.0195750 29694424 PMC5918636

[6] Benevolenza MA, DeRigne LA. The impact of climate change and natural disasters on vulnerable populations: A systematic review of literature. J Health Soc Behav. 2019; 29: 266–281. 10.1080/10911359.2018.1527739

[7] Obradovich N, Migliorini R, Paulus MP, Rahwan I. Empirical evidence of mental health risks posed by climate change. Proc Natl Acad Sci U S A. 2018;115: 10953–10958. 10.1073/pnas.1801528115 30297424 PMC6205461

[8] Wirz-Justice A, Ajdacic V, Rössler W, Steinhausen H-C, Angst J. Prevalence of seasonal depression in a prospective cohort study. Eur Arch Psychiatry Clin Neurosci. 2019; 269: 833–839. 10.1007/s00406-018-0921-3 30022319

[9] Bulbena A, Pailhez G, Aceña R, Cunillera J, Rius A, Garcia-Ribera C, et al Panic anxiety, under the weather? Int J Biometeorol. 2005;49: 238–243. 10.1007/s00484-004-0236-0 15726446

[10] Lee Y-J, Chen Y-T, Ou S-M, Li S-Y, Yang AC, Tang C-H, et al Temperature variation and the incidence of cluster headache periods: A nationwide population study. Cephalalgia. 2014;34: 656–663. 10.1177/0333102413520083 24477598

[11] Yang AC, Fuh J-L, Huang NE, Shia B-C, Wang S-J. Patients with migraine are right about their perception of temperature as a trigger: Time series analysis of headache diary data. J Headache Pain. 2015;16: e49 10.1186/s10194-015-0533-5 PMC4446287 26018293

[12] Ngan S, Toth C. The influence of Chinook winds and other weather patterns upon neuropathic pain. Pain Med. 2011;12: 1523–1531. 10.1111/j.1526-4637.2011.01227.x 21899716

[13] Rifkin DI, Long MW, Perry MJ. Climate change and sleep: A systematic review of the literature and conceptual framework. Sleep Med Rev. 2018;42: 3–9. 10.1016/j.smrv.2018.07.007 30177247

[14] Watson D. Mood and temperament. New York: Guilford Press; 2000.

[15] Connolly M. Some like it mild and not too wet: The influence of weather on subjective well-being. J Happiness Stud. 2013;14: 457–473. 10.1007/s10902-012-9338-2

[16] Lee M, Ohde S, Urayama KY, Takahashi O, Fukui T. Weather and health symptoms. Int J Environ Res Public Health. 2018;15: e1670 10.3390/ijerph15081670 30082669 PMC6122079

[17] Mazza M, Di Nicola M, Catalano V, Callea A, Martinotti G, Harnic D, et al Description and validation of a questionnaire for the detection of meteoropathy and meteorosensitivity: The METEO-Q. Compr Psychiatry 2012;3: 103–106. 10.1016/j.comppsych.2011.02.002 21489419

[18] Sato J, Inagaki H, Kusui M, Yokosuka M, Ushida T. Lowering barometric pressure induces neuronal activation in the superior vestibular nucleus in mice. PLoS ONE. 2019;14: e0211297 10.1371/journal.pone.0211297 30682203 PMC6347159

[19] Žikić M, Rabi-Žikić T. Meteoropathy and meteorosensitive persons. Med Pregl. 2018; 71: 131–135. 10.2298/mpns1804131z

[20] Freti L, Condemi V, Mazza M, Di Nicola M, Janiri L, Antonietti A, et al Meteorosensitivity in a group of patients affected by multiple sclerosis and hospitalized in a rehabilitation facility: An observational study. Altern Integr Med. 2017;6: 252 10.4172/2327-5162.1000252

[21] Akiskal KK, Akiskal HS. The theoretical underpinnings of affective temperaments: Implications for evolutionary foundations of bipolar disorder and human nature. J Affect Disord. 2005;85: 231–239. 10.1016/j.jad.2004.08.002 15780693

[22] DeGeorge DP, Walsh MA, Barrantes-Vidal N, Kwapil TR. A three-year longitudinal study of affective temperaments and risk for psychopathology. J Affect Disord. 2014;164: 94–100. 10.1016/j.jad.2014.04.006 24856560

[23] Rovai L, Maremmani AG, Rugani F, Bacciardi S, Pacini M, Dell'Osso L, et al Do Akiskal & Mallya's affective temperaments belong to the domain of pathology or to that of normality? Eur Rev Med Pharmacol Sci. 2013;17: 2065–2079. 23884828

[24] Blӧink R, Brieger P, Akiskal HS, Marneros A. Factorial structure and internal consistency of the German TEMPS-A scale: Validation against the NEO-FFI questionnaire. J Affect Disord. 2005;85: 77¬83. 10.1016/S0165-0327(03)00101-0 15780678

[25] Oniszczenko W, Stanisławiak E, Dembińska-Krajewska D, Rybakowski J. Regulative Theory of Temperament versus affective temperaments measured by the Temperament Evaluation of Memphis, Pisa, Paris and San Diego Auto-Questionnaire (TEMPS-A): A study in a non-clinical Polish sample. Curr Issues Pers Psychol. 2017;5: 73–82. 10.5114/cipp.2017.65847

[26] Rihmer Z, Akiskal KK, Rihmer A, Akiskal HS. Current research on affective temperaments. Curr Opin Psychiatry. 2010;23: 12–18. 10.1097/YCO.0b013e32833299d4 19809321

[27] Seney ML, Sibille E. Sex differences in mood disorders: perspectives from humans and rodent models. Biol Sex Differ. 2014;5: e17 10.1186/s13293-014-0017-3 PMC4268901 25520774

[28] Vazquez G, Gonda X. Affective temperaments and mood disorders: A review of current knowledge. Curr Psychiatry Rev. 2013;9: 21–32. 10.2174/157340013805289617

[29] Carhart-Harris RL, Nutt DJ. Serotonin and brain function: a tale of two receptors. J Psychopharmacol. 2017;31: 1091–1120. 10.1177/0269881117725915 28858536 PMC5606297

[30] Jenkins TA, Nguyen JCD, Polglaze KE, Bertrand PP. Influence of tryptophan and serotonin on mood and cognition with a possible role of the gut-brain axis. Nutrients. 2016;8: e56 10.3390/nu8010056 45-z 26805875 PMC4728667

[31] Kämpfer S, Mutz M. On the sunny side of life: Sunshine effects on life satisfaction. Soc Indic Res. 2013;110: 579–595. 10.1007/s11205-011-9945-z

[32] Leonard BE. The HPA and immune axes in stress: the involvement of the serotonergic system. Eur Psychiatry. 2005;20 Suppl 3: S302–S306. 10.1016/S0924-9338(05)80180-4 16459240

[33] Walsh MA, Brown LH, Barrantes-Vidal N, Kwapil TR. The expression of affective temperaments in daily life. J Affect Disord. 2013;145: 179–186. 10.1016/j.jad.2012.07.026 22921479 PMC12815226

[34] Amann B, Padberg F., Mergl R, Naber D, Baghai T, Reimers K, et al An investigation of temperamental traits in patients with somatoform disorder: Do they belong in the affective spectrum? Psychosomatics. 2009;50: 605–612. 10.1016/S0033-3182(09)70863-1 19996232

[35] Hyphantis TN, Taunay TC, Macedo DS, Soeiro-de-Souza MG, Bisol LW, Fountoulakis KN, et al Affective temperaments and ego defense mechanisms associated with somatic symptom severity in a large sample. J Affect Disord. 2013;150: 481–489. 10.1016/j.jad.2013.04.043 23706837

[36] Eöry A, Gonda X, Lang Z, Torzsa P, Kalman J Jr, Kalabay L, et al Personality and cardiovascular risk: Association between hypertension and affective temperaments—a cross-sectional observational study in primary care settings. Eur J Gen Pract. 2014; 20: 247–252. 10.3109/13814788.2013.868431 24456347

[37] Oniszczenko W, Rzeszutek M, Stanisławiak E. Affective temperaments, mood, and insomnia symptoms in a non-clinical sample. Behav Sleep Med. 2019;17: 355–363. 10.1080/15402002.2017.1357121 28745523

[38] Ottoni GL, Lorenzi TM, Lara DR (2011) Association of temperament with subjective sleep patterns. J Affect Disord 128:120–127. 10.1016/j.jad.2010.06.014 20584550

[39] Akiskal HS. Toward a definition of generalized anxiety disorder as an anxious temperament type. Acta Psychiatr Scand Suppl. 1998;393: 66–73. 10.1111/j.1600-0447.1998.tb05969.x 9777050

[40] Borkowska A, Rybakowski JK, Drożdż W, Bieliński M, Kosmowska M, Rajewska-Rager A, et al Polish validation of the TEMPS-A: The profile of affective temperaments in a college student population. J Affect Disord. 2010;123: 36–41. 10.1016/j.jad.2009.09.024 19880192

[41] IBM Corp. IBM SPSS Statistics for Windows. Version 25.0. Armonk NY: IBM Corp; 2017.

[42] Tabachnick BG, Fidell LS. Using multivariate statistics.6th ed Boston MA: Pearson; 2013.

[43] Cohen J. Statistical power analysis for the behavioral sciences. 2nd ed Hillsdale NJ: Erlbaum; 1988.

[44] Hayes AF. Introduction to mediation, moderation, and conditional process analysis: A regression-based approach. 2nd ed New York NY: Guilford Press; 2018.

[45] Preacher KJ, Hayes AF (2008) Asymptotic and resampling strategies for assessing and comparing indirect effects in multiple mediator models. Behav Res Methods 40: 879–891. 10.3758/BRM.40.3.879 18697684

[46] Khoury S, Piltonen MH, Ton AT, Cole T, Samoshkin A, Smith SB, et al A functional substitution in the L-aromatic amino acid decarboxylase enzyme worsens somatic symptoms via a serotonergic pathway. Ann Neurol. 2019; 86: 168–180. 10.1002/ana.25521 31177555

[47] Di Nicola M, Mazza M, Panaccione I, Moccia L, Giuseppin G, Marano G, et al Sensitivity to climate and weather changes in euthymic bipolar subjects: Association with suicide attempts. Front Psychiatry. 2020;5: 11–95. 10.3389/fpsyt.2020.00095 PMC7066072 32194448

[48] Mazza M, Mazza O, Pomponi M, Di Nicola M, Padua L, Vicini M, et al What is the effect of selective serotonin reuptake inhibitors on temperament and character in patients with fibromyalgia? Compr Psychiatry. 2009;50: 240–244. 10.1016/j.comppsych.2008.08.004 19374968

[49] Rainville JR, Hodes GE. Inflaming sex differences in mood disorders. Neuropsychopharmacol. 2019;44: 184–199. 10.1038/s41386-018-0124-7 PMC6235877 29955150

[50] Rubinow DR, Schmidt PJ. Sex differences and the neurobiology of affective disorders. Neuropsychopharmacol. 2019;44: 111–128. 10.1038/s41386-018-0148-z PMC6235863 30061743

